# Dual-mode SERS-based lateral flow assay strips for simultaneous diagnosis of SARS-CoV-2 and influenza a virus

**DOI:** 10.1186/s40580-022-00330-w

**Published:** 2022-09-02

**Authors:** Mengdan Lu, Younju Joung, Chang Su Jeon, Sunjoo Kim, Dongeun Yong, Hyowon Jang, Sung Hyun Pyun, Taejoon Kang, Jaebum Choo

**Affiliations:** 1 Department of Chemistry, Chung-Ang University, Seoul, 06974 South Korea; 2 R&D Center, Speclipse Inc., Seongnam, 13461 South Korea; 3 Department of Laboratory Medicine, Gyeongsang National University College of Medicine, Jinju, 52727 South Korea; 4 Department of Laboratory Medicine and Research Institute of Bacterial Resistance, Yonsei University College of Medicine, Seoul, 03722 South Korea; 5 Bionanotechnology Research Center, Korea Research Institute of Bioscience and Biotechnology (KRIBB), Daejeon, 34141 South Korea; 6 School of Pharmacy, Sungkyunkwan University, Suwon, 16419 South Korea

**Keywords:** Surface-enhanced Raman scattering, Lateral flow assay strip, Dual-mode assays, SARS-CoV-2, Influenza a virus

## Abstract

Since COVID-19 and flu have similar symptoms, they are difficult to distinguish without an accurate diagnosis. Therefore, it is critical to quickly and accurately determine which virus was infected and take appropriate treatments when a person has an infection. This study developed a dual-mode surface-enhanced Raman scattering (SERS)-based LFA strip that can diagnose SARS-CoV-2 and influenza A virus with high accuracy to reduce the false-negative problem of the commercial colorimetric LFA strip. Furthermore, using a single strip, it is feasible to detect SARS-CoV-2 and influenza A virus simultaneously. A clinical test was performed on 39 patient samples (28 SARS-CoV-2 positives, 6 influenza A virus positives, and 5 negatives), evaluating the clinical efficacy of the proposed dual-mode SERS-LFA strip. Our assay results for clinical samples show that the dual-mode LFA strip significantly reduced the false-negative rate for both SARS-CoV-2 and influenza A virus.

**Supplementary Information:**

The online version contains supplementary material available at 10.1186/s40580-022-00330-w.

## Introduction

Since the coronavirus disease 2019 (COVID-19) pandemic has continued for a long time, there is a high possibility that the seasonal flu virus is likely to spread simultaneously with severe acute respiratory syndrome coronavirus 2 (SARS-CoV-2) [1, 2]. Therefore, many researchers are rapidly developing a diagnostic method to discriminate between these two respiratory diseases. The initial symptoms of COVID-19 and flu, such as fever, cough, headache, and sore throat, are very similar [3–5]. Thus, it is crucial to quickly identify two viruses for the prevention of viral spread and the appropriate treatment of infected patients. Many molecular diagnostic companies have already commercialized reverse transcription-polymerase chain reaction (RT-PCR) kits to simultaneously diagnose COVID-19 and flu [6, 7]. In addition, colorimetric lateral flow assay (LFA) strips using antibody–antigen interactions have also been commercialized to diagnose these two infectious diseases [8, 9]. The RT-PCR kit enables an accurate diagnosis of COVID-19 and flu but takes a long time to analyze. The LFA strip is fast and straightforward; however, the false-negative issue for the initial infected person is seriously raised due to its poor sensitivity [10–12]. Therefore, it is urgently required to develop a new diagnostic technology that can solve the problems of the long diagnostic time of RT-PCR and the diagnostic accuracy of commercial LFA strips.

A positive diagnosis in a conventional LFA strip is determined by the color change from colorless to red when gold nanoparticles (AuNPs) form sandwich immunocomplexes through antibody–antigen interaction on the test line. However, this visual LFA strip has two critical drawbacks [13–15]. First, false-negative results are frequently produced in diagnosing early infected or asymptomatic patients due to the low detection sensitivity. Second, quantitative analysis is impossible because the naked eyes confirm the color change of the test line. To solve these problems, our research group developed a surface-enhanced Raman scattering (SERS)-based lateral flow assay (LFA) strip that can improve detection sensitivity by 100–1000 times compared to the existing LFA strip. We applied this SERS-LFA strip to detect biomarkers at low concentrations for various diseases [16–20]. In SERS-LFA strips, 40–50 nm Raman reporter-labelled AuNPs (SERS nanotags) were used as detection probes [13]. Furthermore, strong scattering signals could be obtained when the Raman scattering signal is measured by applying a 633 nm He-Ne laser beam into the SERS nanotags [21–25]. Therefore, if SERS-LFA strips are applied to diagnose COVID-19 and flu, it is expected that both the false-negative issue and the problem of quantitative analysis that appear in commercial LFA can be solved. To achieve this goal, we developed dual-mode SERS-LFA strips for simultaneous detection of SARS-CoV-2 and influenza A virus infection.

The proposed dual-mode SERS-LFA strip consists of two test lines and one control line. Capture antibodies that can selectively bind to SARS-CoV-2 and influenza A virus were immobilized on test lines 1 and 2. In addition, detection antibody-conjugated SERS nanotags that can selectively bind to each virus marker flow the strip and form sandwich immunocomplexes at each test line in the presence of target viruses. When the concentration of the target virus is high, a strong SERS signal is displayed, but a weak SERS signal is displayed when the concentration of the target virus is low. This suggests that the amount of each virus can be accurately analyzed by monitoring the characteristic Raman peak intensity of the bound SERS nanotags. To verify the dual-mode SERS-LFA’s performance in a clinical condition, we prepared assay samples by spiking SARS-CoV-2 and influenza A virus lysates in nasopharyngeal samples of normal individuals. Applying this dual-mode SERS-LFA strip is also expected to solve the long measurement time problem, which is the current RT-PCR diagnostic method issue. In addition, it was also possible to significantly reduce the false-negative rate in the commercial LFA strip by substantially enhancing the detection sensitivity. The clinical efficacy of our dual-mode SERS-LFA strip was evaluated by employing 39 patient samples (28 SARS-CoV-2 positives, 6 influenza A virus positives, and 5 negatives). The assay results were compared with RT-PCR and commercial LFA assay data. The proposed dual-mode SERS-LFA strip is expected as a promising diagnostic technology that can quickly and accurately distinguish between SARS-CoV-2 and influenza A virus using a single strip.

## Experimental section

### Reagents and materials

Gold (III) chloride trihydrate (HAuCl_4_·3H_2_O), tris-sodium citrate (Na_3_-citrate), polyvinylpyrrolidone (PVP), and bovine serum albumin (BSA) were purchased from Sigma-Aldrich (St. Louis, MO, USA). 20× Borate buffer (pH 9.0) was purchased from Thermo Fisher Scientific Corporation (Carlsbad, CA, USA). Malachite green isothiocyanate (MGITC) and phosphate-buffered saline (PBS) (10×, pH 7.4) were purchased from Invitrogen Corporation (Carlsbad, CA, USA). Surfactant 10G was purchased from Fitzgerald (Concord, MA, USA). 0.1% Casein blocking agent was purchased from KOMA Biotechnology (Seoul, South Korea). A nucleocapsid protein (N-protein) capture and detection antibodies for SARS-CoV-2 were purchased from Creative Diagnostics (New York, NY, USA). A N-protein capture antibody for influenza A was purchased from Biospacific (Emeryville, CA, USA). A N-protein detection antibody for influenza A was purchased from Abcam (Cambridge, UK). Absorbent pad (CFSP203000) was purchased from Millipore Corporation (Billerica, MA, USA). Influenza A lysate was purchased from Microbix (Mississauga, Canada). SARS-CoV-2 (BetaCoV/Korea/KCDC03/2020, hCoV-19/Korea/KDCA51463/2021) lysate was provided by Korea Research Institute of Bioscience and Biotechnology (KRIBB) (Daejeon, South Korea). Nitrocellulose (NC) membrane (CN110) was purchased from Sartorius (Göttingen, Germany). Backing cards were purchased from Wonkang Bio (Gyeonggido, South Korea). Ninety-six well-plates for enzyme-linked immunosorbent assay (ELISA) were purchased from Corning (Midland, MI, USA). Chung-Ang University Hospital (Seoul, South Korea) provided nasopharyngeal samples of normal individuals.

### Instrumentation

Dynamic light scattering (DLS) data were obtained by Nano-ZS90 (Malvern, UK). UV−visible absorbance of nanoparticles and ELISA data were obtained by a microplate reader (Power Wave ×340, Bio-Tek, VT). The transmission electron microscopy (TEM) images were obtained by a JEM 2100 F of JEOL (Tokyo, Japan). The reagent dispensing system and programmable guillotine cutter were purchased from ZETA Corporation (Gyeonggido, South Korea). Raman mapping images and spectra were obtained by a Renishaw inVia Raman microscope (Renishaw, New Mills, UK) with a He−Ne laser (λ = 632.8 nm) power of 1.0 mW. SERS mapping images were obtained with a 10% laser power and 0.5 s acquisition time under the 20× objective lens. A computer-controlled *x–y* translational stage was scanned in 100 × 100 µm^2^ steps over the *x*-axis of 500 μm and *y*-axis of 2600 μm (total 130 pixels). One hundred and thirty Raman spectra were measured and corrected by the “subtract baseline” function in the WiRE V. 5.3 software (Renishaw, New Mills, UK) to remove the background noises and undesired elements. The intelligent polynomial algorithm performed the baseline fitting (polynomial order = 11). We did not use the smoothing function at this time. The color decoding of Raman mapping profiles at the characteristic Raman peak of MGITC at 1615 cm^− 1^ was also processed by the WiRE V 5.3 software. The scanning electron microscope (SEM) images were obtained by a SEGMA instrument of Carl Zeiss (Oberkochen, Germany). Phase-contrast intensity of a commercial LFA strip was acquired using Image J software.

### Preparation of antibody-conjugated SERS nanotags for SARS-CoV-2 and influenza a virus

AuNPs were synthesized using the seeded-growth method [26]. The concentration of the AuNPs was calculated to be 0.6 nM. Before the modification of AuNPs, the synthesized AuNP solution was diluted to 0.2 nM. Then, 1 µL of 10^− 4^ M MGITC was added to 1.0 mL of AuNP solution. MGITC reporters were attached to AuNPs through two binding forces; one is an Au-S bonding between the isothiocyanate (-NCS), and the other is an electrostatic interaction between MGITC and AuNPs. Because the surface of AuNPs has a negative charge and MGITC is a positively-charged ion, MGITCs could be adsorbed onto the surface of AuNPs by the electrostatic interaction. The mixture was gently stirred at 500 rpm for 30 min at 25 °C. To conjugate detection antibodies on the surface of AuNPs, the pH of MGITC-labeled AuNP solution was kept at 9.0. One hundred microlitre of 50 mM borate buffer was added to the colloidal solution to MGITC-labeled AuNPs to make the surface charge negative. Positively charged antibodies were attached to MGITC-labeled AuNPs via electrostatic interactions. Then 10 µL of 0.3 mg/mL SARS-CoV-2 N-protein antibodies were added to the MGITC-labeled AuNP solution. After shaking (500 rpm) for 30 min, 100 µL of 0.1% casein was added for blocking the unbounded sites on the surface of AuNPs. The solution was stirred for 20 min at 25 °C. The unreacted antibodies and chemicals were removed by centrifugation at 5250 rpm for 15 min, and the supernatant was discarded. The garnet pellets were re-suspended in 100 µL of 5 mM borate buffer. Identical methods were used for the preparation of influenza A virus antibody-conjugated SERS nanotags.

### Fabrication of dual-mode SERS-LFA strips for the simultaneous detection of SARS-CoV-2 and influenza a virus

The dual-mode LFA strip consists of two primary sections: NC membrane and absorbent pad. A plastic backing card was used to hold the two pieces together. Capture antibodies for test and control lines were evenly sprayed onto the NC membrane by a ZETA reagent dispensing system. Here, 0.5 mg/mL anti-SARS-CoV-2 N-protein antibody, anti-influenza A N-protein antibody, and anti-mouse IgG antibody were dispensed on test lines 1, 2, and control line, respectively. The antibody-dispensed NC membrane was allowed to dry for 5 h at 25 °C. The absorbent pad was affixed on the top of the NC membrane. After the cover of the backing card was removed, the NC membrane and absorbent pad were attached with a 2-mm overlap. Then, antibodies were dispensed on the test and control lines of the NC membrane using an automatic dispenser. Lastly, the assembled membrane was sliced into 3.8 mm-wide strips using a programmable guillotine cutter.

### Simultaneous assays of SARS-CoV-2 and influenza a virus using dual-mode SERS-LFA strips

A 96-well plate was used as a medium binding reservoir to simplify the operation process of SERS-LFA. An LFA strip was dipped into an assay mixture-containing well, and the liquid mixtures would flow towards an absorbent pad by capillary force. The solution in the well was prepared by mixing SERS nanotags (3 µL of 2 nM SERS nanotags for SARS-CoV-2 and 3 µL of 2 nM SERS nanotags for influenza A virus), running buffer (4 µL of 5% PVP, 5 µL of 10% surfactant 10 G, 3 µL of 1% gelatin, 1 µL of 1% BSA and 15 µL 1× PBS) and target sample (5 µL of SARS-CoV-2 and 5 µL of influenza A virus in nasopharyngeal samples of normal individuals). The LoDs for calibration curves were calculated by the IUPAC standard using the following equation: LoD = ${Mean}_{blank}$ + 3 S${D}_{blank}$, ${Mean}_{blank}$ is the averaged intensity ratio (I_TL_/I_CL_) of blank, and S${D}_{blank}$is the standard deviation of blank.

### Clinical validation of dual-mode SERS-LFA strips

Total 39 clinical samples (28 SARS-CoV-2 positives, 6 influenza A virus positives, and 5 negatives) were provided from Gyeongsang National University College of Medicine (IRB approval number: 2020-10-002) and Yonsei University Health Service Center, Severance Hospital (IRB Approval number: 4-2020-0465). The clinical assay procedures using SERS-LFA strips were the same as described above.

According to the manufacturer’s instructions, RNA was extracted and purified from clinical samples using the QIAmp Viral RNA Mini kit (Qiagen, Germany). And then, to obtain Ct value (i.e. threshold cycle) of clinical specimens, Luna^®^ Universal One-Step RT-qPCR Kit (NEB, MA, USA) was used in accordance with the manufacturer’s instructions. The fluorescent signal was measured every cycle of PCR step with CFX opus Real-Time System (Bio-Rad, CA, USA), and Ct values were determined by built-in system software (CFX maestro). The sequence of the primer used to target ORF gene of SARS-CoV-2 is as follows. (Forward primer: 5′-TTC TGC TGC TCT TCA ACC TGA-3′, Reverse primer: 5′-ATA GTC TGA ACA ACT GGT GTA AGT-3′). The sequence of the primer used to target M gene of influenza A virus is as follows. (Forward primer: 5′- TCA GGC CCC CTC AAA GCC GA-3′, Reverse primer: 5′-GGG CAC GGT GAG CGT GAA CA-3′).

## Results and discussion

### Detection of SARS-CoV-2 and influenza a virus using colorimetric LFA strips

**Fig. 1:**
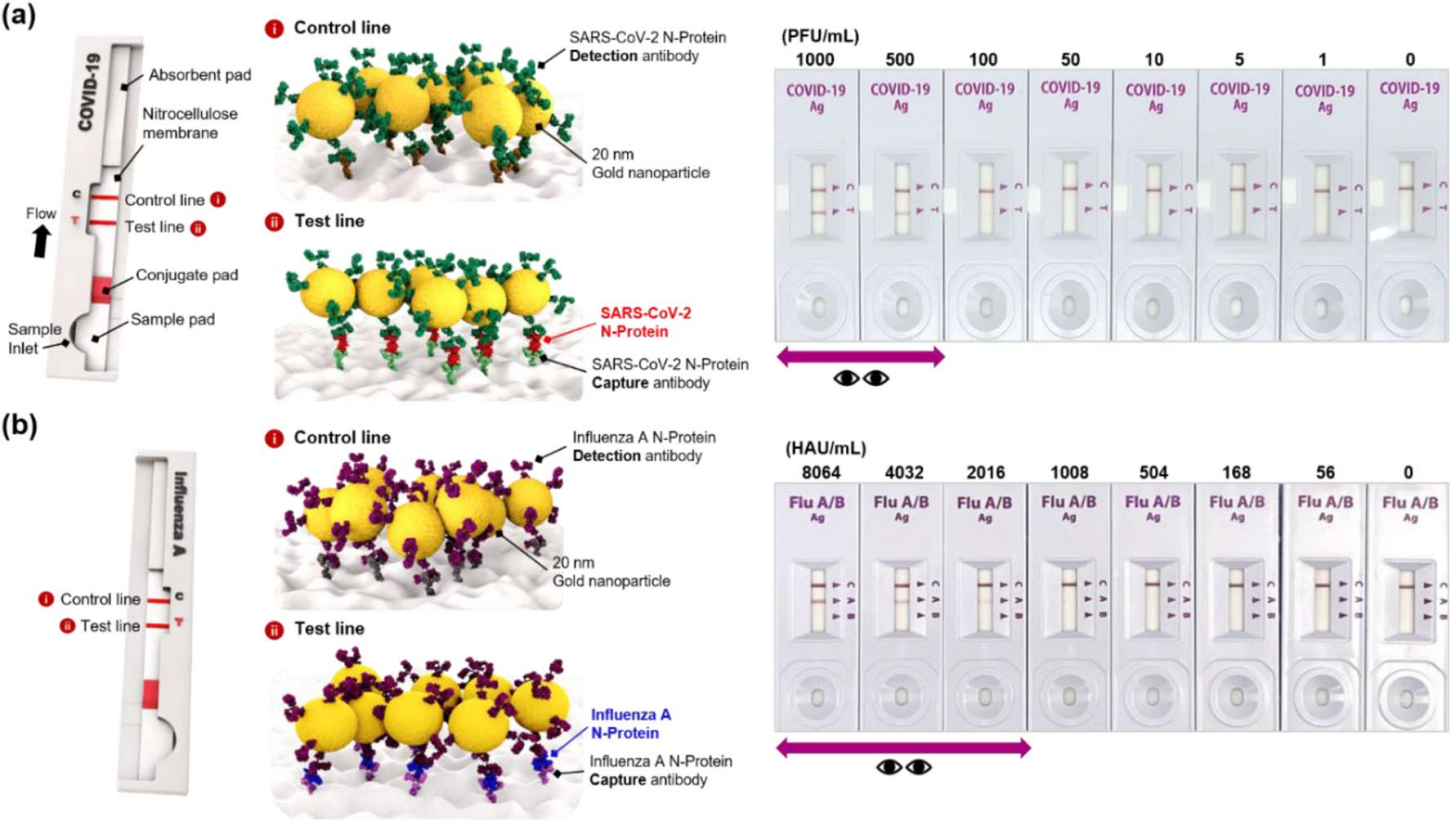
Detection results of SARS-CoV-2 and influenza A virus lysates using commercially available colorimetric LFA strips. **a** Assay results of SARS-CoV-2 lysates along with the increase of SARS-CoV-2 concentration in the 0–1000 PFU/mL range. N-protein in SARS-CoV-2 was used as a target antigen. **b** Assay results of influenza A virus lysates along with the increase of influenza A virus concentration in the 0–8064 HAU/mL range. N-protein in influenza A virus was used as a target antigen

The N-proteins used in this work are suitable as target biomarkers because they are virus genomic RNA-binding proteins that exist largely inside the virus. Most of the commercial LFA strips also use the N-proteins as target biomarkers. Although N-proteins for SARS-CoV-2 and influenza A virus have the same name, they have different binding domains because their structures and amino acid sequences are entirely different [27, 28]. Since the N-proteins of SARS-CoV-2 and influenza A have low cross-reactivity with each other, we chose these proteins to distinguish the two viruses. Figure 1a and b show the detection results of SARS-CoV-2 and influenza A virus lysates using commercially available colorimetric LFA strips from the SD Biosensor. As shown in Fig. 1a, the N-protein target, extracted from SARS-CoV-2, and detection antibody-conjugated AuNPs were sequentially captured by N-protein capture antibodies immobilized on the test line. At this time, the color of the test line changed from colorless to red when SARS-CoV-2 was present. On the other hand, in the absence of SARS-CoV-2, there was no color change because immunocomplexes were not formed on the test line. When various concentrations of SARS-CoV-2 were measured with commercial LFA strips, the limit of detection (LoD) for SARS-CoV-2 was estimated to be approximately 500 plaque-forming unit (PFU)/mL (Fig. 1a). The assays were conducted similarly using commercial LFA strips for influenza A virus. The LFA tests were performed for various influenza A virus concentrations from 0 hemagglutinin unit (HAU)/mL to 8064 HAU/mL. As shown in Fig. 1b, no color change was observed when the concentration of influenza A virus was less than 2016 HAU/mL. Therefore, the LoD of the commercial LFA strip for influenza A was estimated to be approximately 2016 HAU/mL.

When self-diagnosing using commercial LFA strips, it is actually positive but negative in many cases because of the poor sensitivity of the diagnostic strip. In other words, commercial LFA strips appeared to be negative for SARS-CoV-2 in the 1–500 PFU/mL range and influenza A virus in the 1–2016 HAU/mL range, respectively, but they are actually positive. To solve this false-negative diagnostic problem of the commercial LFA strip, a new diagnostic technology that can dramatically improve the detection sensitivity of each virus is essentially required. Therefore, we expected that the dual-mode SERS-LFA strip would significantly reduce the false-negative diagnosis rate by enhancing the detection sensitivity of SARS-CoV-2 and influenza A virus, thereby accurately distinguishing these two respiratory viruses.

### Working principle of dual-mode SERS-LFA strips for simultaneous detection of SARS-CoV-2 and influenza a virus

**Fig. 2:**
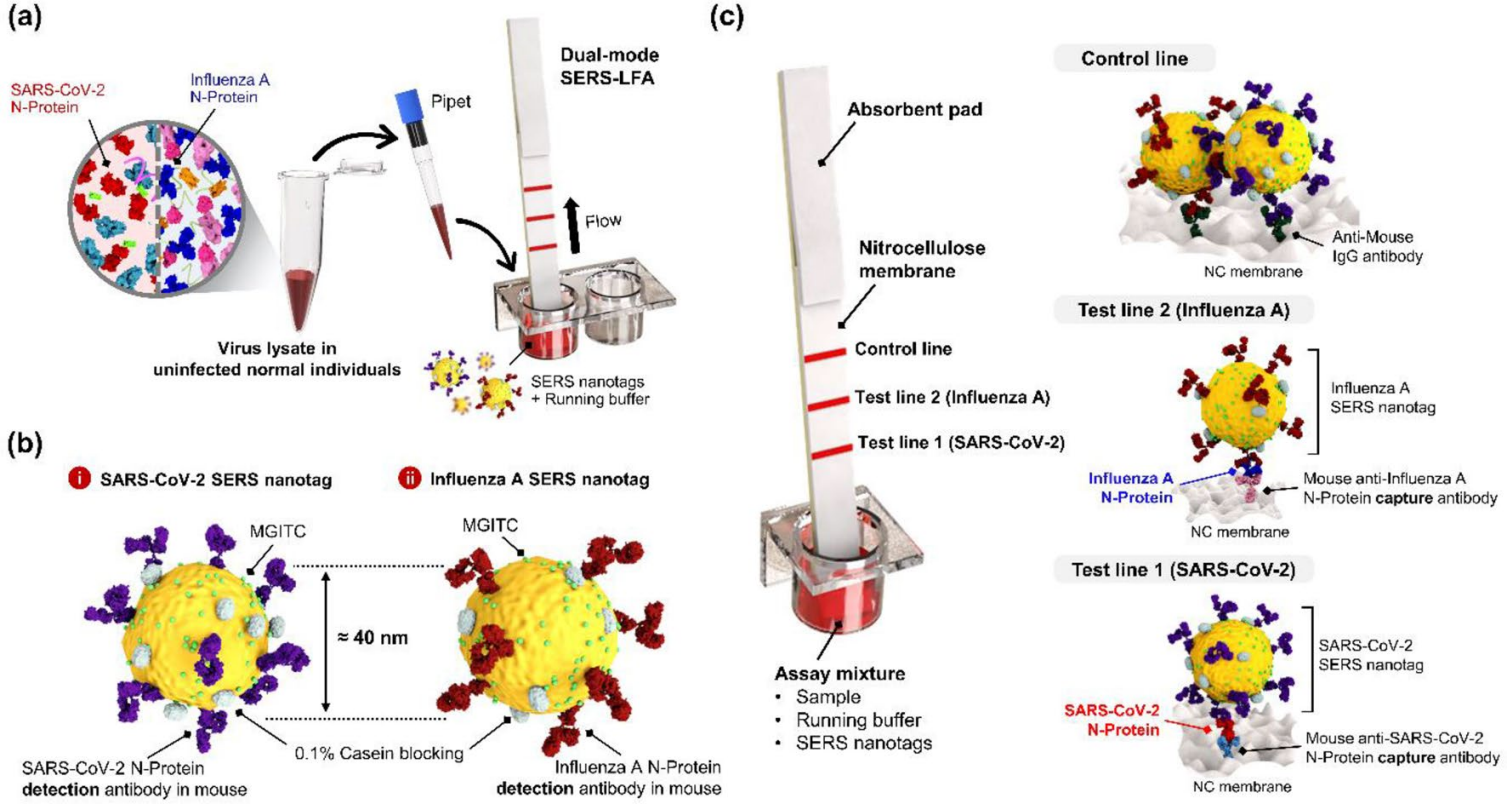
Working principle of the dual-mode SERS-LFA strip for simultaneous detection of SARS-CoV-2 and influenza A virus. **a** Preparation of virus lysates in a nasopharyngeal solution of normal individuals. This virus solution was mixed with SERS nanotags and a running buffer solution in a 96-well plate. **b** N-protein antibody-conjugated SERS nanotags for the detection of SARS-CoV-2 and influenza A virus. **c** SERS nanotags and running buffer move toward the SERS-LFA strip by capillary force. Formation of sandwich immunocomplexes for SARS-CoV-2 (test line 1) and influenza A virus (test line 2)

Figure 2 shows the working principle of the dual-mode SERS-LFA strip for simultaneous detection of SARS-CoV-2 and influenza A virus. A target virus solution was prepared by spiking SARS-CoV-2 and influenza A virus lysates into a nasopharyngeal solution of normal individuals to make conditions similar to the clinical diagnosis process (Fig. 2a). The LFA strip consists of two test lines and one control line. N-protein antibodies for SARS-CoV-2 and influenza A were immobilized on test lines 1 and 2, respectively, and anti-mouse IgG antibodies were immobilized on the control line. We also prepared different concentrations of a target virus solution, detection antibody-conjugated SERS nanotags for SARS-CoV-2 and influenza A virus, and a running buffer solution in an ELISA 96-well plate. When the prepared LFA strip is immersed in each well, the solution in the well moves toward the absorbent pad by capillary force.

Figure 2b shows SERS nanotags for identifying SARS-CoV-2 and influenza A virus. After conjugation of MGITC Raman reporters and N-protein detection antibodies on AuNPs, 0.1% casein was used as a blocking agent to stabilize the remaining area of the AuNP surface. Additional file 1: Fig. S1 shows TEM images of (a) AuNPs and (b) SERS nanotags, and corresponding (c) DLS distributions and (d) UV–vis absorption spectra. TEM images show that AuNPs have uniform size distributions. Since antibodies or Raman reporter molecules attached to the surface of AuNPs do not appear in the TEM image, we confirmed their attachment through DLS distribution (Additional file 1: Fig. S1c) and UV–vis spectra (Additional file 1: Fig. S1d). According to the DLS distribution, the average diameter of AuNPs and SERS nanotags increased from 50 to 68 nm, and the UV–vis absorption maximum was red-shifted from 530 to 535 nm. Therefore, we could confirm that SERS nanotags were successfully synthesized from DLS and UV–vis spectral data. As shown in Fig. 2c, SERS nanotags and target N-proteins form sandwich immunocomplexes on test line 1 in the presence of SARS-CoV-2 lysates. On the other hand, sandwich immunocomplexes are formed on test line 2 when influenza A virus lysates are present. Additional file 1: Fig. S2 shows SEM images for each test line in the presence and absence of viruses. In both cases, it was observed that many nanoparticles were bound to each test line by the interaction between SERS nanotags and N-proteins when the viruses were present. SERS nanotags are always captured on the control line through antibody–antibody interactions regardless of the presence of viruses. After the flow through the LFA strip is finished, SERS signals were collected on test and control lines to evaluate SARS-CoV-2 and influenza A virus quantitatively.

### Simultaneous detection of SARS-CoV-2 and influenza a virus using dual-mode SERS-LFA strips

**Fig. 3:**
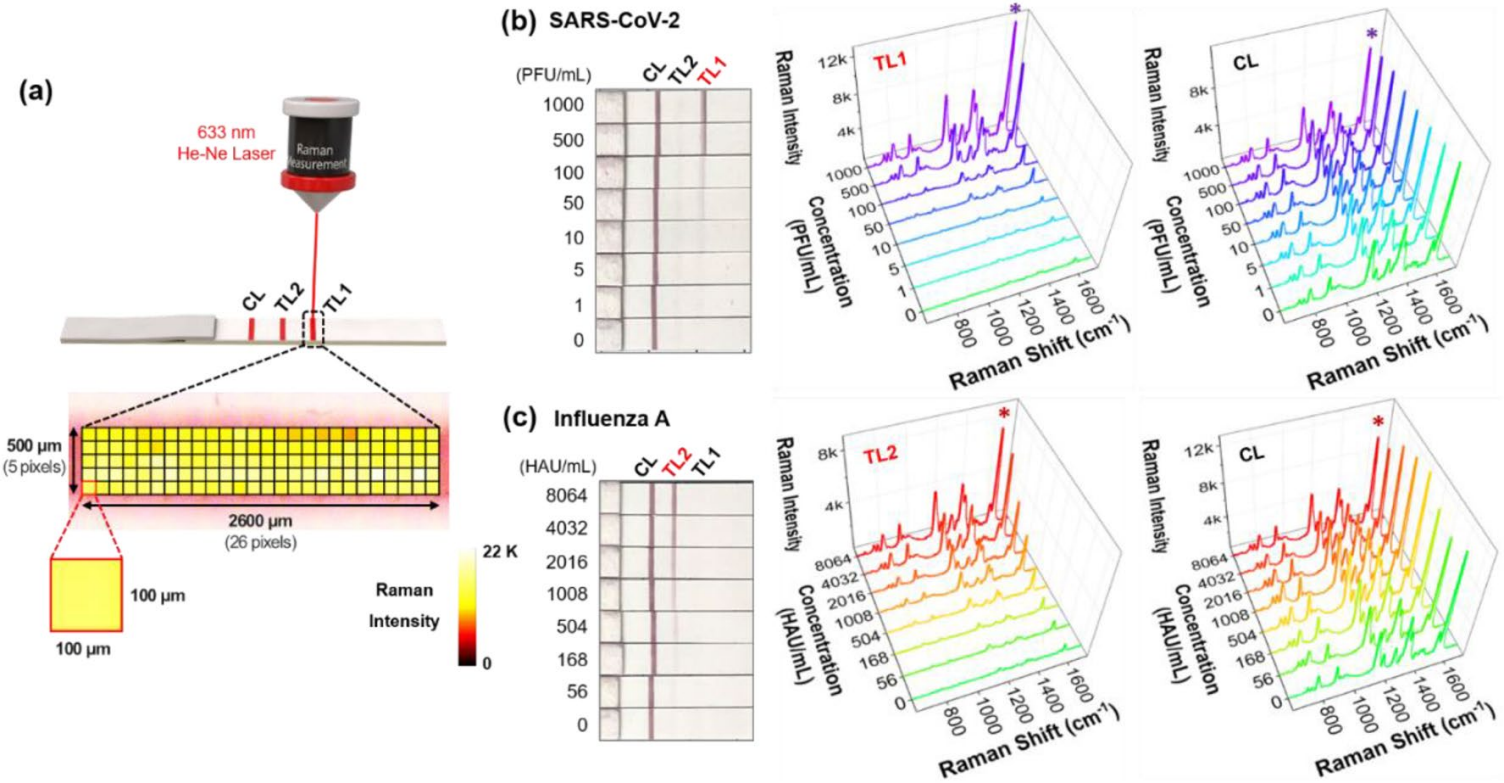
**a** Pixel-to-pixel SERS detections for an area of 2600 μm × 500 μm with a 100 μm interval for test and control lines. Raman signal intensities for 130 spots were averaged to obtain reliable Raman signal intensity. **b** Visual color changes of the SERS-LFA strip and corresponding SERS spectra of the test and control lines when the concentration of SARS-CoV-2 lysate changed in the range of 0–1000 PFU/mL. **c** Visual color changes of the SERS-LFA strip and corresponding SERS spectra of the test and control lines when the concentration of influenza A virus lysate changed in the range of 0–8064 HAU/mL

In SERS-based assays, it is critical to secure the reproducibility that is formed from localized surface plasmon effects. Therefore, multiple SERS spectra were measured and averaged. As shown in Fig. 3a, pixel-to-pixel detections for an area of 2600 μm × 500 μm with a 100 μm interval were sequentially performed for test and control lines. Then the Raman signal intensities for 130 (26 × 5) pixels were baseline-subtracted to obtain reliable Raman signal intensity in Additional file 1: Fig. S3. In this way, we evaluated the analytical performance of the dual-mode SERS-LFA strip by changing the concentration of one virus type only. The color change of the SERS-LFA strip and corresponding SERS spectra of the test and control lines were measured when the concentration of SARS-CoV-2 lysate changed in the range of 0–1000 PFU/mL as shown in Fig. 3b. According to the photographic images of the SERS-LFA strips, we can see a certain intensity of red color, indicating that the control line is correctly operating. On the other hand, test line 1 for SARS-CoV-2 showed an apparent color change at a concentration of 500 PFU/mL and more, but a color change at the concentration lower than that was difficult to observe. Test line 2 for influenza A virus showed no color change at this time.

In the case of influenza A virus, on the contrary, test line 1 for SARS-CoV-2 showed no color change at all, and test line 2 showed an evident color change at a concentration of 1008 HAU/mL and more (Fig. 3c). These experimental results show that the dual-mode SERS-LFA strip has a specific selectivity for SARS-CoV-2 and influenza A virus. Nevertheless, naked-eye identification has a false-negative diagnostic problem for an infected person with a low concentration of SARS-CoV-2 or influenza A virus. Virus assays using dual-mode SERS-LFAs were performed to reduce this false-diagnostic rate. Raman spectra were averaged after measuring 130 pixels for test and control lines (Fig. 3b and c). In both SARS-CoV-2 and influenza A virus cases, the SERS intensity decreases consistently as the virus concentration decreases. On the other hand, the control lines’ SERS intensities were constant in both cases. Our experimental results indicate that the SERS-LFA strips can simultaneously detect SARS-CoV-2 and influenza A virus with high sensitivity.

### Performance evaluation of dual-mode SERS-LFA strips for detection of SARS-CoV-2 and influenza a virus

**Fig. 4:**
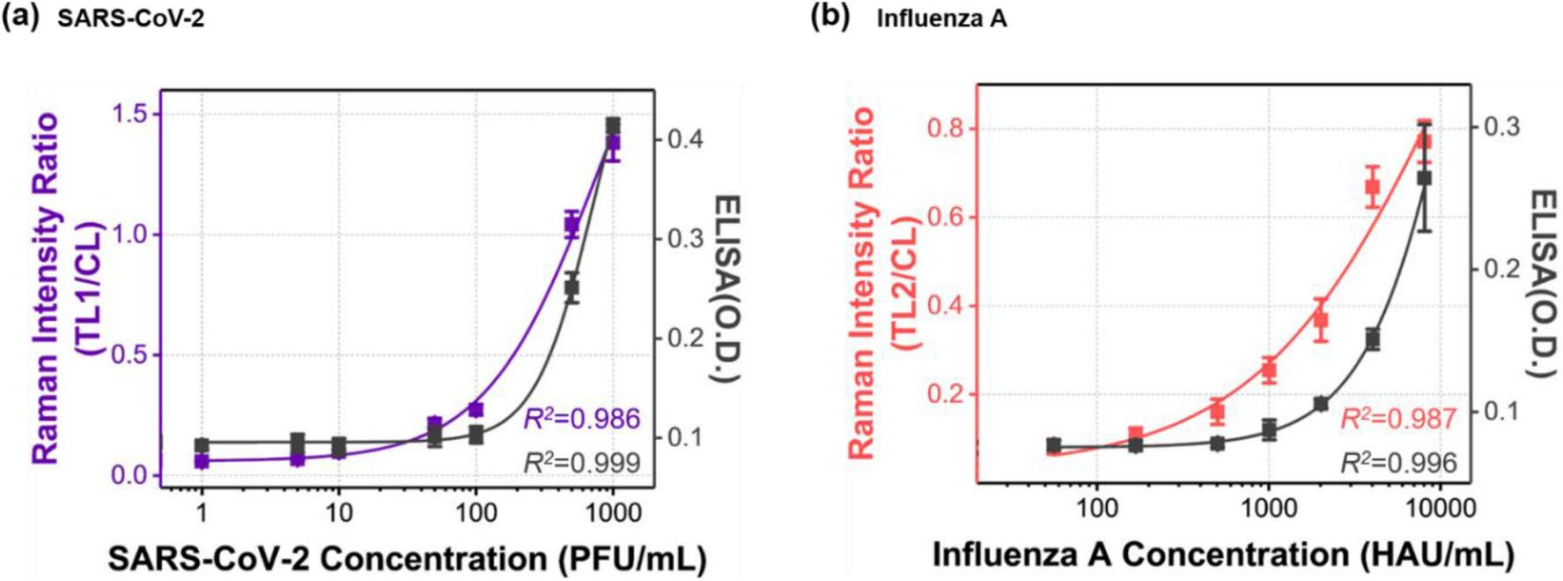
Comparison of calibration curves for **a** SARS-CoV-2 and **b** influenza A virus, determined by the measurement results of ELISA and dual-mode SERS-LFA. Each calibration curve was determined using a four-parameter sigmoidal fitting equation (black: ELISA, purple and red: SERS-LFA). The *y-*axis represents the optical density for ELISA and the Raman scattering intensity ratio of the test and control lines for SERS-LFA

The calibration curves for SARS-CoV-2 and influenza A virus, determined by the measurement results of ELISA and SERS-LFA, are compared in Fig. 4. The characteristic Raman peak intensity at 1615 cm^− 1^ of MGITC was used for quantitative analysis of both viruses. Each calibration curve was determined using a four-parameter sigmoidal fitting equation. The *y*-axis represents the optical density for ELISA and the scattering intensity ratio of the test line and control line for SERS-LFA. In both ELISA and SERS-LFA, virus concentration and optical signal intensity showed a good correlation. For SARS-CoV-2 and influenza A virus, each LoD was determined from calibration curve data measured by ELISA and SERS-LFA.

**Fig. 5:**
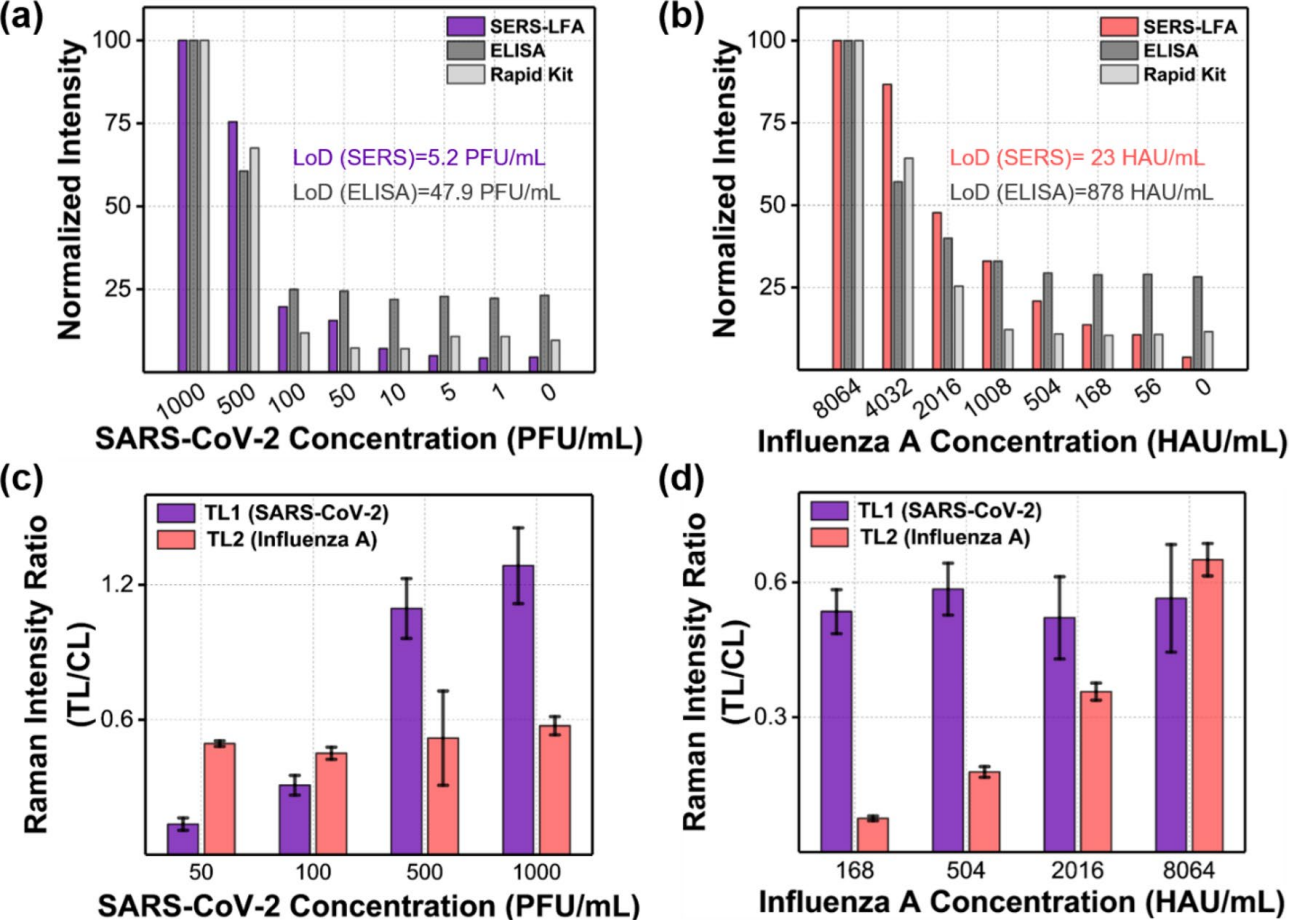
Cross-reactivity test results of the dual-mode SERS-LFA strip against SARS-CoV-2 and influenza A virus. Normalized intensity variations of SERS-LFA (Raman peak intensity at 1615 cm^− 1^), ELISA (absorbance intensity), and colorimetric LFA (phase contrast intensity) for **a** SARS-CoV-2 (0–1000 PFU/mL) and **b** influenza A virus (0–8064 HAU/mL). **c** SERS intensity ratio variations of test lines 1 and 2 when the SARS-CoV-2 concentration was changed in the range of 50–1000 PFU/mL, but the concentration of influenza A virus was fixed at 8064 HAU/mL. **d** SERS intensity ratio variations of test lines 1 and 2 when the influenza A virus concentration was changed in the 168–8064 HAU/mL range, but the concentration of SARS-CoV-2 was fixed at 200 PFU/mL

In Fig. 5a and b, we compared LoD values ​​for the commercial colorimetric strip, ELISA, and dual-mode SERS-LFA strip for SARS-CoV-2 and influenza A virus. The intensity values were normalized based on the highest concentration of each virus. In the case of SARS-CoV-2, LoDs were determined to be 48 PFU/mL for ELISA and 5.2 PFU/mL for SERS-LFA strip, respectively. The SERS-LFA strip exhibits approximately 10 times better sensitivity than ELISA. For the colorimetric LFA strip, LoD was 500 PFU/mL (Fig. 1a). In the case of influenza A virus, LoDs were estimated to be 880 HAU/mL for ELISA and 23 HAU/mL for SERS-LFA strip, proving that the SERS-LFA strip has approximately 40 times better sensitivity than ELISA. The LoD of the colorimetric LFA strip was 1008 HAU/mL (Fig. 1b). Overall, the dual-mode SERS-LFA strip has a good selectivity against SARS-CoV-2 and influenza A virus. It is also more sensitive than the colorimetric LFA strip and ELISA assay currently used for immunoassays of these viruses. Furthermore, using the SERS-LFA strip, it is possible to distinguish between SARS-CoV-2 and influenza A virus.

Figure 5c and d show the cross-reactivity test results of the dual-mode SERS-LFA strip against SARS-CoV-2 and influenza A virus. SERS intensities of test lines 1 and 2 were measured when the concentration of SARS-CoV-2 was changed in the range of 50−1000 PFU/mL, but the concentration of influenza A virus was fixed at 8064 HAU/mL (Fig. 5c). The SERS intensity for SARS-CoV-2 in test line 1 increased consistently as the concentration increased in the 50−1000 PFU/mL range while the SERS intensity for influenza A virus was kept constant at 8064 HAU/mL (Fig. 5c). Conversely, when the concentration of influenza A virus was changed in the range of 168–8064 HAU/mL, but the concentration of SARS-CoV-2 was kept constant at 200 PFU/mL, only the SERS intensity of influenza A virus was changed along with its concentration (Fig. 5d). These experimental results show that the SARS-CoV-2/influenza A virus dual-mode LFA strip developed in this study has good selectivity for each virus. Additionally, a selectivity test for five different respiratory viruses was performed using a dual-mode SERS-LFA strip. As shown in Additional file 1: Fig. S4, our dual-mode SERS-LFA strip showed good selectivity for only influenza A virus (H1N1 and H3N2 types) and SARS-CoV-2 among the five viruses.

### Clinical validation of dual-mode SERS-LFA strips for detection of SARS-CoV-2 and influenza a virus

Table 1 shows clinical assay results using dual-mode SERS-LFA strips for 39 patient samples (28 SARS-CoV-2 positives, 6 influenza A virus positives, and 5 negatives). RT-PCR results for ORF1 of SARS-CoV-2 were used as control data [29, 30]. SARS-CoV-2 positive samples were classified into four levels (< 20, 20–25, 25–30, and > 30) according to the Ct levels. It is known that clinical samples in Ct < 20 have a high virus concentration. On the other hand, clinical samples in Ct > 30 need more thermo-cycling amplification steps, which means that the virus concentration is relatively low. We compared the assay results tested by the dual-mode SERS-LFA strips with those tested by commercial SARS-CoV-2 and influenza A virus LFA strips to evaluate the diagnostic efficacy of the proposed dual-mode SERS-LFA strips. For SARS-CoV-2, both LFA and SERS-LFA strips showed positive results for one clinical sample in the Ct < 20 levels. For the seven samples in the 20 < Ct < 25 levels, six (85%) and seven (100%) samples showed positive for LFA and SERS-LFA, respectively. For the nine samples in the 25 < Ct < 30 levels, six (67%) and eight (88%) clinical samples showed positive for LFA and SERS-LFA, respectively. Finally, for the 11 samples in the Ct > 30 levels, one (9%) and six (60%) showed positive for LFA and SERS-LFA, respectively. In the case of influenza A virus, four out of six clinical samples showed positive (66%) for LFA, but all the six samples showed positive (100%) for SERS-LFA. Both LFA and SERS-LFA showed all negative (100%) for five negative clinical samples.

Additional file 1: Table S1 shows the SERS intensity ratios (TL1/CL) for eight different SARS-CoV-2 concentrations. The corresponding calibration curve (Fig. 4a) was determined by the four-parameter sigmoidal fitting equation in Additional file 1: Table S3. Here, the LoD of TL1/CL for SARS-CoV-2 was determined to be 0.062, and the corresponding concentration was 5.2 PFU/mL. Therefore, if the TL1/CL value measured for the clinical sample was greater than 0.062, it was determined as positive, and if it was less than 0.062, it was determined as negative. Positive/negative discrimination was performed on 33 SARS-CoV-2 samples, and the results are listed in Table 1. Positive/negative discrimination for influenza A virus was also performed similarly to SARS-CoV-2. Additional file 1: Table S2 shows the SERS intensity ratios (TL2/CL) for eight different influenza A virus concentrations. The corresponding calibration curve (Fig. 4b) was determined by the four-parameter sigmoidal fitting equation in Additional file 1: Table S3. The LoD of TL2/CL for influenza A virus was determined to be around 0.048, and the corresponding concentration was 23 HAU/mL. Positive/negative discrimination was performed on 11 influenza virus A samples, and the results are listed in Table 1.

**Table 1:** Clinical assay results for SARS-CoV-2 and influenza A virus using RT-PCR, commercial LFA, and dual-mode SERS-LFA strips performed on 39 patient samples

Sample	No.	Ct value (ORF1 RNA)	Group	LFA (P/N)	Dual-mode SERS-LFA		
					Ratio (TL1/CL)		P/N
SARS-CoV-2 Positive	P1	18.87	< 20	P	1.601		P
	P2	22.78	20–25	P	0.215		P
	P3	22.9		P	0.285		P
	P4	23.47		P	0.54		P
	P5	23.9		N	0.096		P
	P6	24.59		P	0.209		P
	P7	24.68		P	1.138		P
	P8	24.95		P	0.107		P
	P9	25.12	25–30	P	0.113		P
	P10	25.83		P	0.133		P
	P11	26.57		P	0.236		P
	P12	26.65		P	0.123		P
	P13	26.87		N	0.064		P
	P14	27.34		P	0.072		P
	P15	29.52		N	0.067		P
	P16	29.69		P	0.292		P
	P17	29.7		N	0.012		N
	P18	30.07	> 30	P	0.077		P
	P19	30.45		N	0.142		P
	P20	30.77		N	0.029		N
	P21	31.25		N	0.042		N
	P22	32.38		N	0.022		N
	P23	32.83		N	0.108		P
	P24	33.09		N	0.075		P
	P25	33.88		N	0.018		N
	P26	33.96		N	0.027		N
	P27	34.85		N	0.080		P
	P28	35.92		N	0.145		P

Sample	No.	Ct value ( M RNA)	Group	LFA (P/N)	Dual-mode SERS-LFA		
					Ratio (TL2/CL)		P/N
Influenza A virus Positive	P1	25	25–30	P	0.073		P
	P2	25.67		P	0.082		P
	P3	25.67		P	0.335		P
	P4	27.67		N	0.230		P
	P5	32.67	> 30	P	0.099		P
	P6	33.67		N	0.082		P

Sample	No.	Ct value	Group	LFA (P/N)	Dual-mode SERS-LFA		
					Ratio (TL1/CL)	Ratio (TL2/CL)	P/N
Negative	N1	N/A	Negative	N	0.018	0.016	N
	N2	N/A		N	0.029	0.027	N
	N3	N/A		N	0.027	0.012	N
	N4	N/A		N	0.022	0.017	N
	N5	N/A		N	0.011	0.027	N

Table 2 summarizes the positive/negative discrimination results based on the SARS-CoV-2/influenza A virus assays performed by commercial LFA and our dual-mode SERS-LFA strips on 39 clinical samples. Overall, in the assays using dual-mode SERS-LFA strips, the false-negative rate was significantly reduced compared to commercial LFA strips. In particular, the false-negative rate was reduced considerably in clinical samples with Ct > 25, which is due to the high sensitivity of the SERS-LFA strip. Although a sufficient number of clinical samples could not be tested for influenza A virus, the false-negative rate was also significantly reduced, like SARS-CoV-2 when dual-mode SERS-LFA strips were used for the clinical assays. In conclusion, when using the dual-mode SERS-LFA assay strips developed in this study, the false-negative rate was remarkably reduced compared to individual SARS-CoV-2 or influenza A virus LFA strips.

**Table 2:** Statistical analysis of the diagnostic results of SARS-CoV-2 and influenza A virus using LFA and dual-mode SERS-LFA strips on 39 clinical samples

Sample	Group	Sensitivity	
		LFA	Dual-mode SERS-LFA
SARS-CoV-2 Positive	< 20	100% (1/1)	100% (1/1)
	20–25	85% (6/7)	100% (7/7)
	25–30	66% (6/9)	88% (8/9)
	> 30	9% (1/11)	54% (6/11)
Influenza A virus Positive	25–30	75% (3/4)	100% (4/4)
	> 30	50% (1/2)	100% (2/2)

Sample	Group	Specificity	
		LFA	Dual-mode SERS-LFA
Negative	Negative	100% (5/5)	100% (5/5)

## Conclusion

In this study, we developed a dual-mode SERS-LFA strip that can quickly and accurately diagnose which virus was the patient infected among SARS-CoV-2 and influenza A virus. Using this dual-mode SERS-LFA strip could overcome the poor sensitivity and limitations of quantitative analysis of existing colorimetric LFA strips. Further, the problem of reproducibility of the SERS detection technology could be solved by measuring and averaging successive SERS signals for multiple spots on the test and control lines. N-protein antibodies immobilized on test lines 1 and 2 for SARS-CoV-2 and influenza A virus showed good selectivity to the lysates of each virus. When SARS-CoV-2 and influenza A virus were simultaneously assayed using this SERS-LFA strip, their corresponding LoD was estimated to be 5.2 PFU/mL and 23 HAU/mL, respectively. These values were 10 times and 40 times more sensitive than the ELISA measurement results. Clinical analysis data on 39 patients’ samples (28 SARS-CoV-2 positive, 6 influenza A virus positive, 5 negative) showed that dual-mode SERS-LFA strips could significantly reduce the false-negative rate in commercially available colorimetric LFAs for diagnosis of SARS-CoV-2 and influenza A virus. Furthermore, we could simultaneously identify two different respiratory diseases, SARS-CoV-2 and influenza A virus, using a single strip.

## Supplementary Information

**Additional file 1: Fig. S1.** TEM images of (a) AuNPs and (b) SERS nanotags. (c) DLS size distributions, (d) corresponding UV–vis absorption spectra of AuNPs (black) and SERS nanotags (red). **Fig. S2.** SEM images for test lines: (a) in the absence (0 PFU/mL) and presence (1000 PFU/mL) of SARS-CoV-2, and (b) in the absence (0 HAU/mL) and presence (8064 HAU/mL) of influenza A virus. **Fig. S3.** Raman spectra of 130 mapping points before (gray) and after (blue/red) base line corrections for (a) 1000 PFU/mL SARS-CoV-2 and 8064 HAU/mL influenza A virus. **Fig. S4.** Selectivity tests for five different respiratory viruses (RSV, influenza B, influenza A/H3N2, influenza A/H1N1, and SARS-CoV-2) using a SERS-LFA strip. **Table S1.** SERS intensity ratios for eight different SARS-CoV-2 concentrations. **Table S2.** SERS intensity ratios for eight different influenza A virus concentrations. **Table S3.** Determination of LoDs using four-parameter sigmoidal function and calculated LoD values for ELISA and dual-mode SERS-LFA.

## Data Availability

The datasets used and/or analyzed during the current study are available from the corresponding author on reasonable request.
