# Supplementary material for: Dual-mode SERS-based lateral flow assay strips for simultaneous diagnosis of SARS-CoV-2 and influenza a virus
Source: Nano Converg. 2022 Sep 2;9:39. doi: 10.1186/s40580-022-00330-w (PMC9441817; doi:10.1186/s40580-022-00330-w)
Supplement: Supplementary file 1 — Additional file 1: Fig. S1. TEM images of (a) AuNPs and (b) SERS nanotags. (c) DLS size distributions, (d) corresponding UV–vis absorption spectra of AuNPs (black) and SERS nanotags (red). Fig. S2. SEM images for test lines: (a) in the absence (0 PFU/mL) and presence (1000 PFU/mL) of SARS-CoV-2, and (b) in the absence (0 HAU/mL) and presence (8064 HAU/mL) of influenza A virus. Fig. S3. Raman spectra of 130 mapping points before (gray) and after (blue/red) base line corrections for (a) 1000 PFU/mL SARS-CoV-2 and 8064 HAU/mL influenza A virus. Fig. S4. Selectivity tests for five different respiratory viruses (RSV, influenza B, influenza A/H3N2, influenza A/H1N1, and SARS-CoV-2) using a SERS-LFA strip. Table S1. SERS intensity ratios for eight different SARS-CoV-2 concentrations. Table S2. SERS intensity ratios for eight different influenza A virus concentrations. Table S3. Determination of LoDs using four-parameter sigmoidal function and calculated LoD values for ELISA and dual-mode SERS-LFA. [file 40580_2022_330_MOESM1_ESM.docx]

***Additional Information***

**Dual-mode SERS-based lateral flow assay strips for simultaneous diagnosis of SARS-CoV-2 and influenza A virus**

Mengdan Lu^1,a^, Younju Joung^1,a^, Chang Su Jeon^2^, Sunjoo Kim^3^, Dongeun Yong^4^, Hyowon Jang^5^, Sung Hyun Pyun^2,*^, Taejoon Kang^5,*^, Jaebum Choo^1,*^

^1^ *Department of Chemistry, Chung-Ang University, Seoul 06974, South Korea*

^2^ *R&D Center, Speclipse Inc., Seongnam 13461, South Korea*

^3^ *Department of Laboratory Medicine, Gyeongsang National University College of Medicine, Jinju 52727, South Korea*

^4^ *Department of Laboratory Medicine and Research Institute of Bacterial Resistance, Yonsei University College of Medicine, Seoul 03722, South Korea*

^5^ *Bionanotechnology Research Center, Korea Research Institute of Bioscience and Biotechnology (KRIBB), Daejeon 34141, South Korea*


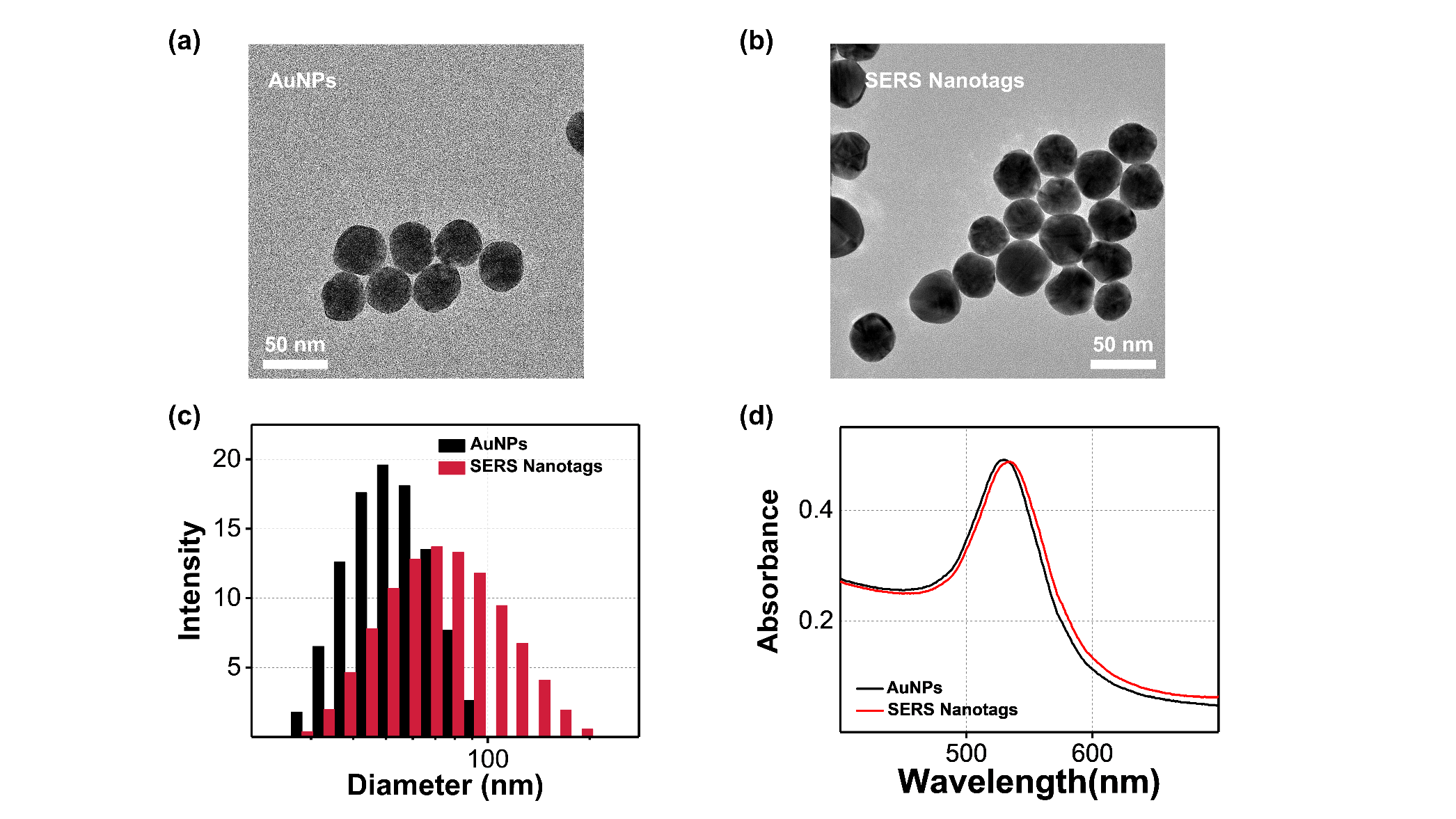


**Fig. S1**. TEM images of (a) AuNPs and (b) SERS nanotags. (c) DLS size distributions, (d) corresponding UV-vis absorption spectra of AuNPs (black) and SERS nanotags (red).


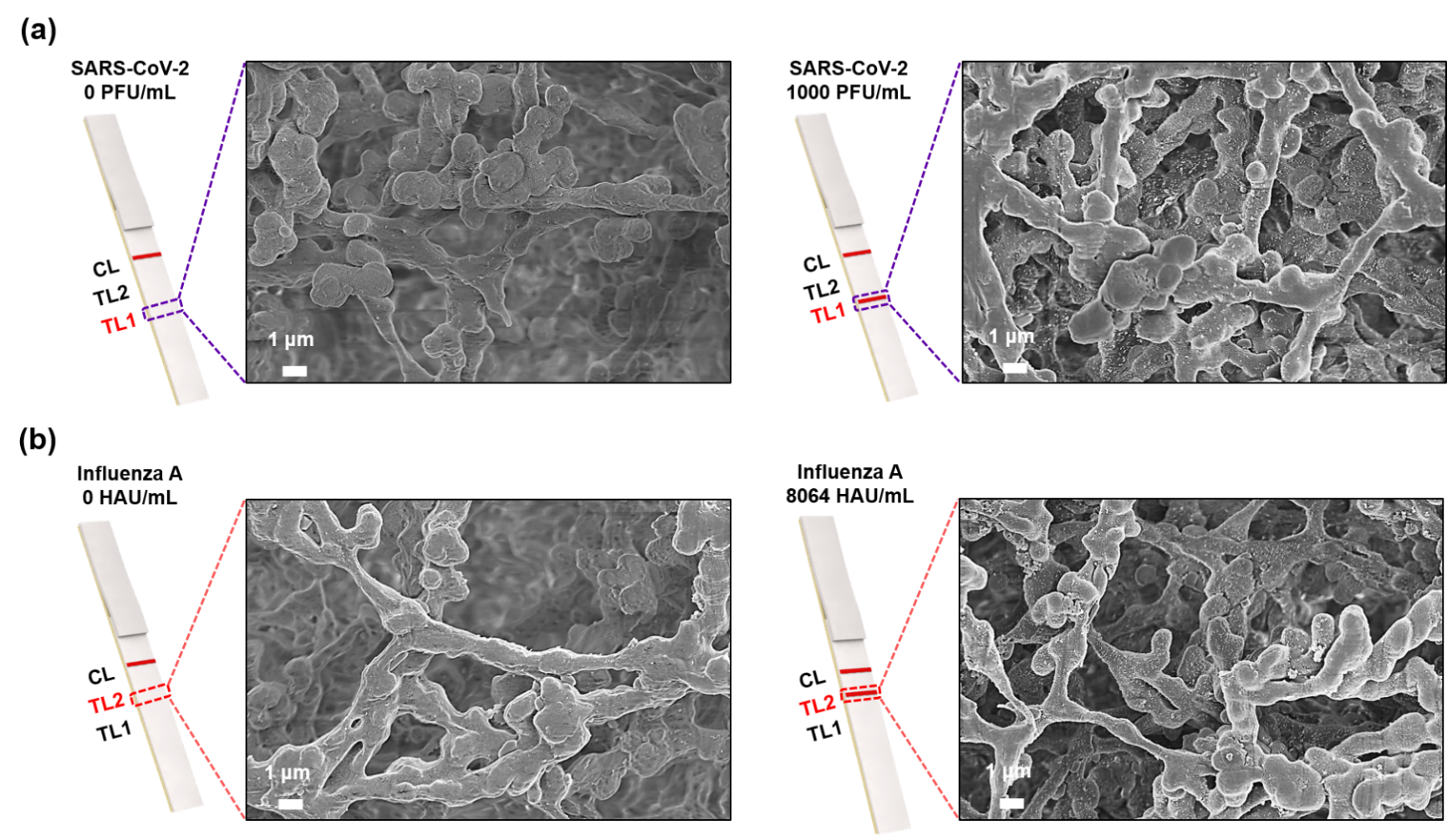


**Fig. S2**. SEM images for test lines: (a) in the absence (0 PFU/mL) and presence (1000 PFU/mL) of SARS-CoV-2, and (b) in the absence (0 HAU/mL) and presence (8064 HAU/mL) of influenza A virus.


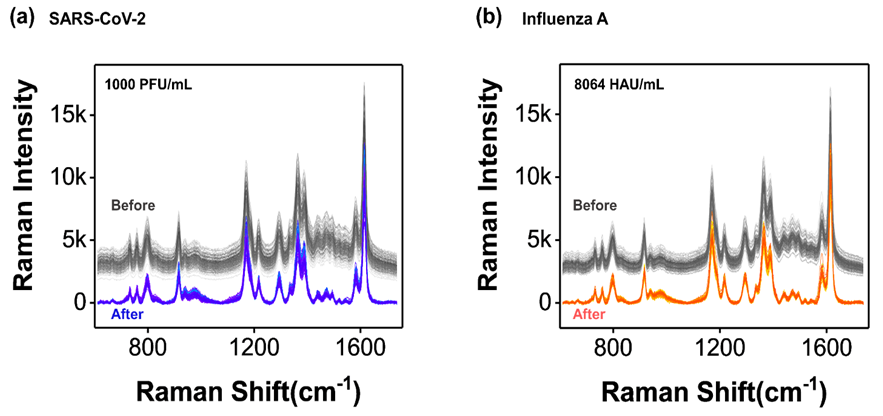


**Fig. S3.** Raman spectra of 130 mapping points before (gray) and after (blue/red) base line corrections for (a) 1000 PFU/mL SARS-CoV-2 and 8064 HAU/mL influenza A virus.


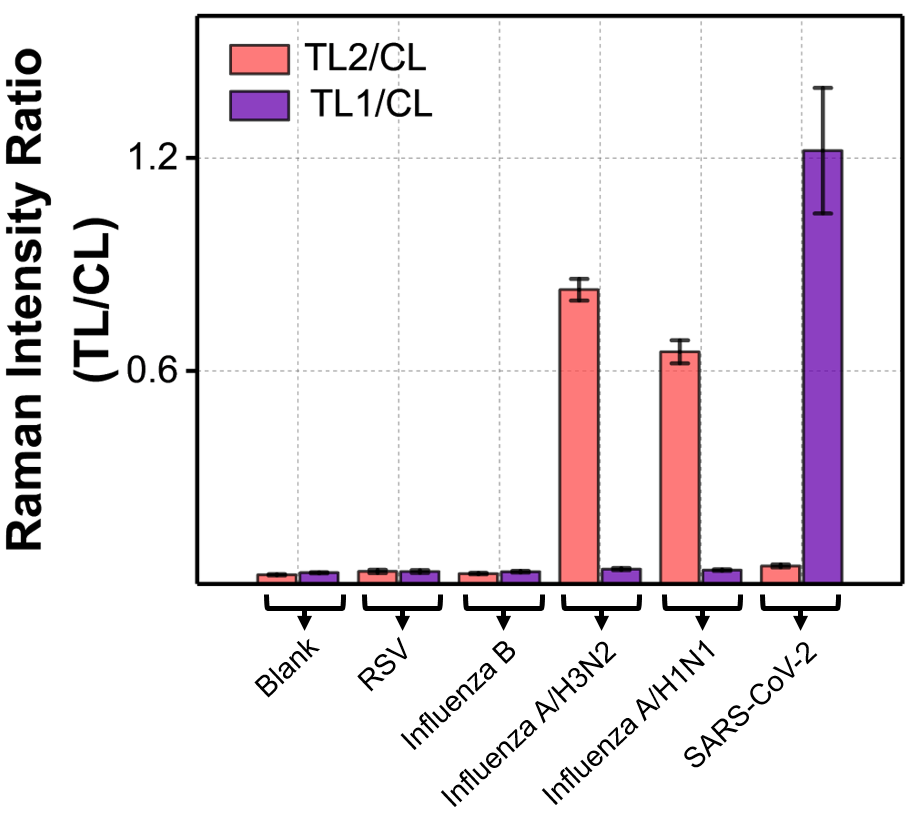


**Fig. S4**. Selectivity tests for five different respiratory viruses (RSV, influenza B, influenza A/H3N2, influenza A/H1N1, and SARS-CoV-2) using a SERS-LFA strip.

**Table S1.** SERS intensity ratios for eight different SARS-CoV-2 concentrations.

| Concentration (PFU/mL) | 1000 | 500 | 100 | 50 | 10 | 5 | 1 | | 0 |
| --- | --- | --- | --- | --- | --- | --- | --- | --- | --- |
| TL1 | 12978.3 | 10458.4 | 2879.09 | 2230.93 | 861.36 | 734.36 | | 657.40 | 600.08 |
| CL | 9395.64 | 10036.1 | 10551.9 | 10370.2 | 8763.94 | 10694.8 | | 11194.6 | 11540.2 |
| Ratio (TL1/CL) | 1.381 | 1.042 | 0.273 | 0.215 | 0.098 | 0.068 | | 0.058 | 0.052 |

**Table S2.** SERS intensity ratios for eight different influenza A virus concentrations.

| Concentration (HAU/mL) | 8064 | 4032 | 2016 | 1008 | 504 | 168 | 56 | | 0 |
| --- | --- | --- | --- | --- | --- | --- | --- | --- | --- |
| TL2 | 7806.26 | 7113.04 | 4748.64 | 3456.06 | 2244.07 | 1292.90 | | 938.26 | 671.16 |
| CL | 10137.6 | 10644.36 | 2937.67 | 13607.2 | 14169.5 | 12389.7 | | 11483.5 | 22906.5 |
| Ratio (TL2/CL) | 0.770 | 0.668 | 0.367 | 0.254 | 0.158 | 0.104 | | 0.082 | 0.029 |

**Table S3.** Determination of LoDs using four-parameter sigmoidal function and calculated LoD values for ELISA and dual-mode SERS-LFA.

| **Methods** | **Model** | **Equation** | **Parameters** | |
| --- | --- | --- | --- | --- |
|  |  |  | **Term** | **Value** |
| ELISA  (SARS-CoV-2) | Four-parameter sigmoidal function | $y=\frac{A_{1}-A_{2}}{1+{(x/{x_{0})}}^{p}}+A_{2}$ | *A_1_* | 0.09579 |
|  |  |  | *A_2_* | 0.57348 |
|  |  |  | *x_0_* | 713.13 |
|  |  |  | *p* | 2.0520 |
| ELISA  (Influenza A virus) |  |  | *A_1_* | 0.07502 |
|  |  |  | *A_2_* | 0.88293 |
|  |  |  | *x_0_* | 18502 |
|  |  |  | *p* | 1.4707 |
| Dual-mode SERS-LFA  (SARS-CoV-2) |  |  | *A_1_* | 0.05980 |
|  |  |  | *A_2_* | 2.51215 |
|  |  |  | *x_0_* | 807.95 |
|  |  |  | *p* | 1.0412 |
| Dual-mode SERS-LFA  (Influenza A virus) |  |  | *A_1_* | 0.03143 |
|  |  |  | *A_2_* | 1.9543 |
|  |  |  | *x_0_* | 13759 |
|  |  |  | *p* | 0.74507 |
